# Hospital falls prevention with patient education: a scoping review

**DOI:** 10.1186/s12877-020-01515-w

**Published:** 2020-04-15

**Authors:** Hazel Heng, Dana Jazayeri, Louise Shaw, Debra Kiegaldie, Anne-Marie Hill, Meg E. Morris

**Affiliations:** 1 La Trobe Centre for Sport and Exercise Medicine Research, La Trobe University, Bundoora, Australia; 2 Healthscope and Faculty of Health Science, Youth & Community Studies, Holmesglen Institute, Melbourne, Australia; 3 School of Physiotherapy and Exercise Science, Faculty of Health Sciences, Curtin University, Perth, Western Australia Australia; 4 Victorian Rehabilitation Centre, ARCH Healthscope and La Trobe Centre for Sport and Exercise Medicine Research, La Trobe University, Bundoora, Australia

**Keywords:** Falls, Prevention, Injury, Physiotherapy, Patient education, Hospital, Healthcare

## Abstract

**Background:**

Hospital falls remain a frequent and debilitating problem worldwide. Most hospital falls prevention strategies have targeted clinician education, environmental modifications, assistive devices, hospital systems and medication reviews. The role that patients can play in preventing falls whilst in hospital has received less attention. This critical review scopes patient falls education interventions for hospitals. The quality of the educational designs under-pinning patient falls education programmes was also evaluated. The outcomes of patient-centred falls prevention programs were considered for a range of hospital settings and diagnoses.

**Methods:**

The Arksey and O’Malley (2005) framework for scoping reviews was adapted using Joanna Briggs Institute and PRISMA-ScR guidelines. Eight databases, including grey literature, were searched from January 2008 until February 2020. Two reviewers independently screened the articles and data were extracted and summarised thematically. The quality of falls prevention education programs for patients was also appraised using a modified quality metric tool.

**Results:**

Forty-three articles were included in the final analysis. The interventions included: (i) direct face-to-face patient education about falls risks and mitigation; (ii) educational tools; (iii) patient-focussed consumer materials such as pamphlets, brochures and handouts; and (iv) hospital systems, policies and procedures to assist patients to prevent falls. The included studies assessed falls or education related outcomes before and after patient falls prevention education. Few studies reported incorporating education design principles or educational theories. When reported, most educational programs were of low to moderate quality from an educational design perspective.

**Conclusions:**

There is emerging evidence that hospital falls prevention interventions that incorporate patient education can reduce falls and associated injuries such as bruising, lacerations or fractures. The design, mode of delivery and quality of educational design influence outcomes. Well-designed education programs can improve knowledge and self-perception of risk, empowering patients to reduce their risk of falling whilst in hospital.

## Background

Falls in hospitals remain an ongoing concern, despite world-wide recognition of this persistent problem [1]. Rates vary widely across hospitals globally and typically range from 3 to 11 falls per 1000 bed days [2–4]. Around 25% of hospital falls are injurious, and result in fractures, soft-tissue injuries and fear of falling [5–7]. As reported in the National Institute for Health and Care Excellence (NICE) guidelines [8], hospitalised older adults are at risk of falling due to factors such as ill health, co-morbidities, anaesthetics, pain, medications, polypharmacy and muscle weakness, yet many patients do not realise their risk [9–12]. Patient education is one strategy to address this gap by increasing engagement in falls prevention programs [6, 13]. Alongside clinician education, medication management, multi-disciplinary reviews, environmental modifications, assistive devices, and hospital systems and policies, education assists patients to self-manage their own falls risk [6, 14–17].

Patient education is important because there can be a mismatch between perceived and actual falls risk whilst in hospital [10–12]. Hospital falls risks have been historically assessed using tools such as the Falls Risk Assessment Tool (FRAT) [18], St Thomas’s Risk Assessment Tool (STRATIFY) [19] and the Hendrich II Fall Risk Model [20]. Some of these assign a falls risk score to each patient [18–20]. Clinicians can also use their clinical judgement and application of research evidence on a case-to-case basis to determine falls risk. Carefully considered, evidence-based decision making can assist the selection of individualised falls prevention interventions [21]. Unfortunately, some patients appear to engage in risk taking behaviours that increase their falls risk [22] such as not pressing the call bell when needing to walk to the toilet [23], or not waiting for nurses to arrive before attempting to mobilise, when they are unsafe to walk without supervision [12]. Particularly for people with poor balance, cognitive impairment or gait disorders, there is an increased falls risk whilst in hospital [12]. Up to 80% of falls occur when patients are not observed [24]. Some patients initiate risky decisions about mobility based on their own judgements, without always seeking help from nurses or other health professionals [25]. Others report feeling secure by virtue of being in a hospital environment, even though they are actually at high risk of falling [11, 22, 26]. Although risk taking is not always problematic, it becomes dangerous when excessive, poorly considered or not in a supported environment [22].

Patient education aims to increase a person’s awareness of their own falls risk and to provide them with strategies to mitigate falls whilst hospitalised [27]. There are varying levels of evidence for different methods of patient falls prevention education, such as handouts [28, 29], videotapes [30, 31], posters [32, 33], falls risk communication alerts and assistive devices (such as sensors, wristbands and bed alarms) [33, 34], and face-to-face discussions about safe footwear and other interventions [35, 36]. Whilst education is an aspect of most hospital falls prevention programs, few studies have evaluated the outcomes or design of educational components, based on educational theory [14]. A systematic review by Lee et al. [37] reported preliminary evidence for the benefits of delivering hospital patient education informed by educational theory and the principles of health behaviour change. Recent investigations add further weight to the idea that falls mitigation interventions that incorporate evidence-based design are successful at reducing falls [38–42].

For these reasons, we conducted a scoping review to identify gaps in current research by summarising and evaluating different sources of evidence from systematic reviews, narrative literature reviews, clinical trials and grey literature [41, 42]. Given the potential for patient education to mitigate hospital falls, this scoping review aimed to (i) conduct an up to date search of hospital falls prevention interventions pertaining to patient education; (ii) appraise the design of hospital patient education programs and; (iii) identify and critique variables, tools and measures used to quantify changes in falls and associated outcomes.

## Methods

### Protocol and registration

The protocol for this scoping review incorporates frameworks developed by Arksey and O’Malley [42], the Joanna Briggs Institute [43] and the Preferred Reporting Items for Systematic Reviews and Meta-analysis Extension for Scoping Reviews (PRISMA-ScR) [44]. The methods were described in detail in a published protocol [45].

### Research question

The broad research question was "What are the findings of current literature regarding patient falls prevention education in hospitals?" [45] The specific questions were: (i) what was the content of the patient education program? (ii) what mode of delivery was used? (iii) was the design informed by educational principles or evidence-based behaviour change models? (iv) what were the main outcomes?

### Identifying relevant studies

#### Eligibility criteria

Studies were included if they investigated hospital falls prevention interventions that utilised patient education. Educational interventions designed for the families of cognitively impaired hospitalised patients were also included. Educational interventions that were delivered in the emergency department with the intent of only reducing falls post-discharge were excluded. Also excluded were investigations outside of the hospital setting or in paediatric populations. Non-empirical reports were excluded. Any studies that were directed towards clinician education alone were excluded. All study designs were included, such as quantitative, qualitative and mixed-methods designs, to ensure that the full breadth of literature was captured. Only English language studies were included.

#### Search

An initial limited search of PubMed and Cumulative Index to Nursing and Allied Health Literature (CINAHL) was conducted to identify key words and index terms. A qualified librarian established a search strategy consisting of key words and index terms, together with medical subject headings (MeSH) related to falls prevention and patient education in hospitals. This search strategy was conducted across Allied and Complementary Medicine Database (AMED), PubMed, the Cochrane Central Register of Controlled Trials (CENTRAL), PsychINFO, CINAHL and Education Resources Information Center (ERIC) for articles published from January 2008 to February 2020. Trove and ProQuest Theses and Dissertations Global were searched for grey literature. Finally, reference lists of selected articles for full text review were hand searched. An example search strategy is in Additional file 1.

#### Study selection

The results from the searches were uploaded into Covidence® (a web platform for systematic reviews) and duplicates were identified and removed. The title and abstracts of articles were screened by two independent reviewers (HH, LS). The same reviewers obtained and assessed the full text of identified papers. Any discrepancies were discussed and resolved through consensus.

### Data charting

A data extraction chart was developed to identify the key characteristics of each study as well as relevant information regarding the characteristics of patient falls education. Two reviewers independently charted the data and resolved inconsistencies through discussion with a third researcher. The variables included authors, publication year, country of origin, aims, settings falls prevention methods, patient characteristics, education program characteristics, measurement tools and reported outcomes.

The quality of the patient falls education program for each study was assessed by an independent researcher (HH) using a quality metric (Additional file 2). Any uncertainties were discussed with a second independent research who also reviewed the articles, until consensus was reached (DJ). We adapted the tool created by Kiegaldie and Farlie [46] which assesses the quality of education programs in falls prevention research for health professionals. The modified metric excludes clinician education specific items and identifies components of the education program such as the aim and setting, characteristics of the learner and teacher, learning activities and evaluation of the program. The scoring system assigned one point to each ‘yes’ response and no points to each response that was ‘no’ or ‘not stated’. A total of 0–6 points was considered to represent low quality, 7–12 points to represent moderate quality and 13–17 to indicate high quality.

### Collating, summarising and reporting the results

The data extracted were summarised using thematic analysis [45]. The studies were grouped by their design, characteristics of education interventions and outcomes. When a systematic review was identified, the studies reported in that review were screened and added if they met the inclusion criteria and were not already incorporated into the search yield. As documented in our protocol paper [45], systematic reviews were included to summarise the highest level of evidence in current literature, in addition to narrative reviews, clinical research trials and grey literature.

## Results

### Study characteristics

The search of the databases yielded a total of 9340 citations. The flow of studies is presented as a PRISMA flow chart (Additional file 3). Following full text review, 73 records were excluded. Five systematic and three non-systematic reviews were identified, and their references searched, providing an additional five articles to be included. A total of 43 articles were finally included in the current review (Additional file 4). They were from the USA, Australia, Israel, Singapore, China, Japan, Switzerland, the Netherlands and Canada. The highest proportion of studies was from the USA (*n* = 24).

Of the included articles, five were systematic reviews (Table 1) [14, 37, 47–49], three were targeted literature reviews [6, 50, 51], 10 were randomised controlled trials (RCTs) (Table 2) [30–32, 34, 38, 52, 53, 55, 57], three were qualitative studies [58–60], and three were unpublished theses [61–63]. The remaining studies were quasi-experimental trials (*n* = 18). Several of these utilised the same overall data set yet reported different outcomes. Haines et al. [64] examined economic evaluations of falls prevention programs following an RCT [30]. Three qualitative studies reported staff [58], educator [59] and participant perspectives [60] following an intervention carried out in an earlier study [38]. For the purposes of this scoping review, investigations such as these were analysed together.

**Table 1:** Systematic review characteristics

Lead author (Year)	Aim	Studies reviewed (n)	Hospital RCTs (n)	Inpatient education single intervention RCTs (n)	Inpatient multifactorial interventions with patient education RCTs (n)	Method of assessing quality	Quality of evidence	Conclusion
Avanecean (2017) [47]	Assess effectiveness of patient-centered falls prevention in acute care	5	5	0	3	JBI-SUMARI critical appraisal tool	Multifactorial: High	Multifactorial: May reduce falls rate Meta-analysis not performed
Cameron (2018) [14]	Evaluate effectiveness of falls prevention for older adults in hospitals and care facilities	95	24	2	6	GRADE	Patient education alone: Very low Multifactorial: Low	Patient education: Unable to conclude effectiveness No pooled rate ratio Multifactorial: May reduce falls rate RR: 0.80 (0.64–1.01)
Hempel (2013) [48]	Review characteristics and effectiveness of falls prevention in acute care	59	4	0	1	Egger regression and Begg rank rest	No evidence of publication bias	Multifactorial: Unable to conclude effectiveness RaR: 0.77 (0.52–1.12)
Lee (2014) [37]	Evaluate the effectiveness of patient falls education in hospitals and post discharge	26	16	5	8	Law tool	Patient education alone: Moderate Multifactorial: Moderate	Patient education alone or in multifactorial: Significant reduction in falls rate RR: 0.77 (0.69–0.87)
Miake-Lye (2013) [49]	Evaluate effectiveness of multifactorial falls prevention programs in hospital and factors related to successful implementation	21	21	0	12	Downs and Black Quality Score	Multifactorial: Moderate	Multifactorial: May reduce falls rate Meta-analysis not performed

Footnote: Mutifactorial refers to two or more of the following: patient education, falls risk assessments, environmental modifications, devices, personal supervision, multidisciplinary reviews, medication reviews, falls risk communication aids, allied health and nursing input, rounding, staff training

*RCT* Randomised controlled trial, *JBI-SUMARI* Joanna Briggs Institute System for the Unified Management, Assessment and Review of Information, *GRADE* Grading of Recommendations, Assessment, Development and Evaluation, *RR* Risk ratio, *RaR* Rate ratio

**Table 2:** Randomised controlled trials characteristics

Lead author (Year)	Setting	Interventions	Education content	Education delivery modes	Education design guiding principles	Education outcomes	Education quality	Fall outcomes
Aizen (2015) [52]	Sub-acute	Multifactorial v Usual Care	Behavioural and cognitive treatment with patient and family guidance	Not stated	Not stated	Not reported	2/17 Low	No difference in falls (falls per 1000 bed days: experimental 1; control 2) (*p* = 0.11)
Ang (2011) [34]	Acute	Multifactorial v Usual Care	Falls education based on individual falls risk	Face to face by nurses	Not stated	Not reported	6/17 Low	Less falls in experimental group than control group (*p* = 0.018)
Cumming (2008) [53]	Acute, sub-acute	Multifactorial v Usual Care	Falls education based on individual falls risk	Face to face by nurses	Not stated	Not reported	5/17 Low	No difference in falls (falls per 1000 bed days: experimental 9; control 9)
Dykes (2010) [54]	Acute	Multifactorial v Usual Care	Falls education based on individual falls risks	Handout	Yes. Handout designed to match consumer literacy	Not reported	4/17 Low	Less falls in experimental than control group (falls per 1000 bed days: experimental 3; control 4) (*p* = 0.04)
Haines (2011) [22]	Acute, sub-acute	Group 1: Combination Group 2: Materials Group 3: Usual Care	Education on falls, self-reflection of individual risk, falls strategies, goal setting	Face to face by physiotherapist Handout given by trained clinician Combination of all	Yes. Content based on health belief model and consumer feedback	Not reported	7/17 Moderate	No difference in falls (falls per 1000 bed days: combination 8; materials 8; control 9)
Hill (2009) [55]	Acute, sub-acute	Education of patient delivered by video v Education of patient delivered by handout	Education on risk of falls and falls prevention strategies	Handout Video	Yes. Content based on health belief model and utilising design and communication principles	Yes. Video group identified more falls prevention strategies than handout group (*p* = 0.02). Video group was more motivated and confident to reduce falls than handout group (*p* = 0.03)	8/17 Moderate	No falls outcome reported
Hill (2015) [38]	Sub-acute	Education of patient v Usual Care	Education on falls, cues to action and goal setting	Face to face plus handout plus video	Yes. Content based on health belief model and adult learning principles	Not reported	11/17 Moderate	Less falls in experimental group than control group (falls per 1000 bed days: experimental 8; control 14) (*p* = 0.03)
Kuhlenschmidt (2016) [31]	Acute	Education of patient v Usual Care	Education on fall risks, strategies and fear of falling, tailored to different risk categories	Face to face plus handout plus video by research nurses	Not stated	Yes. Risk perception changed more in the intervention group (*p* = 0.01)	11/17 Moderate	No falls outcomes reported
Kiyoshi-Teo (2019) [56]	Acute	Education of patient v Usual care	Education on fall risks, strategies and prompting behaviour change and self-reflection of falls prevention	Face to face plus handout by research nurse	Yes. Content based on motivational interviewing concept	Yes. No significant difference between groups in confidence, falls prevention behaviours and patient engagement	10/17 Moderate	No significant difference in falls (incidence rates per month: experimental 0.2029; control 0.2098)
van Gaal (2011) [57]	Acute	Multifactorial v Usual Care	Education on falls prevention	Face to face plus handout given by nurses	Not stated	Not reported	2/17 Low	Less falls in experimental group than control group (rate ratio 0.67)

Footnote: Mutifactorial refers to two or more of the following: patient education, falls risk assessments, environmental modifications, devices, personal supervision, multidisciplinary reviews, medication reviews, falls risk communication aids, allied health and nursing input, rounding, staff training

Most of the trials were in an acute hospital setting (*n* = 22). Seven were conducted in sub-acute settings [36, 38, 52, 58–60, 65] and six included both acute and sub-acute hospital wards [30, 53, 55, 64, 66, 67]. Six studies reported including cognitively impaired patients [30, 36, 38, 52, 53, 65]. Only Haines et al. and Hill et al. conducted sub-group analyses of cognitively impaired patients [30, 38].

Less than half of the studies trialled patient education as a single intervention (*n* = 11) [28–31, 35, 38, 55, 56, 61–63]. The remaining investigations reported multi-factorial falls prevention programs that included a patient education component. Broadly, the falls education interventions could be categorised as (i) direct education of the patient about falls mitigation via face-to-face discussions that could include the use of videotapes [28–31, 33–36, 38, 52, 53, 55, 58, 61–64, 66, 68–75]; (ii) educational tools such as posters on walls to prevent falls [32, 33, 67, 69, 76]; (iii) consumer materials such as pamphlets and written guides for patients and families [28–33, 35, 38, 54–58, 61, 62, 64, 65, 68, 70–72, 74–76]; and (iv) hospital falls prevention systems, policies or procedures that included an element of patient education [32, 33, 36, 54].

### Content of patient falls education programs

Most hospital patient education programs focused on providing information to individuals about falls risks, and how to prevent falling (Additional file 5). Some only reported educating patients about risk whilst in hospital [29, 33]. Others reported educating patients more generally about falls prevention strategies at home, in the community and whilst in hospital [36, 67, 72, 76]. These instructions varied between studies. Several delivered individualised patient education based on risk factors that were identified through risk assessments [32, 34, 53, 54, 63, 73, 74]. Examples included using the call bell when needing to transfer, waiting for the nurses to arrive to assist with mobility, encouragement to use appropriate footwear and instructions about using appropriate assistive devices to walk [32, 34, 53, 54, 63, 73, 74]. Two studies incorporated goal setting within patient education [30, 38]. Another two reported engaging the patient to identify their own falls risk [30, 33]. Some reports did not provide enough description of the content of the education program to enable replication of exact elements [30, 52, 57, 69, 70, 75].

### Education delivery mode

A range of modes of patient falls education were used, with some investigators implementing combinations of approaches (Additional file 5). The most prevalent mode was face-to-face education, where either hospital clinicians or research staff delivered falls education directly to the patient [34–36, 53, 66, 73]. Others used videotapes [62], handouts [32, 54, 57, 65] or fall prevention posters [67] as single modalities. Some combined face to face discussions with handouts [28, 29, 56, 68, 71, 74, 75], videotapes [61, 63], posters [69, 72], or a combination of videotapes and handouts [30, 31, 38]. Yet others combined handouts with videotaped education [55, 70], posters [76], or with posters and videotapes [33].

### Educational design principles and models

The majority of articles in this review did not provide any information on educational design, theoretical models or guiding principles on which programs were based (Additional file 5) [28, 31, 33–36, 52, 53, 57, 61, 63, 65–76]. Three reported the content of the education program to be designed with reference to the Health Belief Model [30, 38, 55]. The Health Belief Model poses that health behaviour change occurs when an individual’s perception of threats, barriers, benefits and self-efficacy are acknowledged and managed [77]. Three studies designed falls education programs with consideration of adult learning principles [29, 33, 38], which posits that adults learn best when they have high self-esteem, are active in knowledge gain, receive appropriate feedback and have low levels of anxiety [78]. Others reported taking into consideration the level of consumer literacy [32, 54], theoretical principles of educational design and communication [55], or principles of patient engagement when designing the falls education program [62]. Three investigations used the teach-back method to assess what the patient learned. Based on this assessment they targeted gaps in knowledge [35, 61, 73]. Another study engaged patients with motivational interviewing to encourage behaviour change to prevent falls [56]. Such educational design principles reflected the recommendations of the Prevention of Falls Network Europe (ProFaNE) guidelines, which advocate increasing awareness of falls risk, positive self-identity, and self-management [79].

### Education and fall outcomes

#### Education-specific outcomes

Educational outcomes were reported in six trials [29, 31, 54–56, 63]. Cerilo et al. [63], Dykes et al. [54], Hill et al. [55] and Kuhlenschmidt et al. [31] assessed patient self-perceived ratings of their falls risk. Cerilo et al. used the Falls Risk Awareness Questionnaire (FRAQ) which is yet to report validity and reliability [80]. Hill et al. and Kuhlenschmidt et al. both developed questionnaires to evaluate knowledge, motivation and satisfaction [31, 55]. Kuhlenschmidt et al. reported validating their questionnaire readability, accuracy, adaptability and reliability [31]. Dykes et al. rated patient self-perceived falls risk using a Likert scale [54]. All of these investigators noted that self-perceived falls risk improved post falls-prevention intervention. Hill et al., Huang et al. and Kuhlenschmidt et al. also showed that patient education was effective in increasing knowledge about predisposing factors to slips, trips and falls [29, 31, 55].

Additional outcomes included patient confidence and fear of falling. Cerilo et al., Kiyoshi-Teo et al. and Huang et al. measured patient confidence in completing daily activities together with fear of falling [29, 56, 63]. Cerilo et al. and Kiyoshi-Teo et al. used the Falls Efficacy Scale (FES) and Falls Efficacy Scale International-Short (FESI-S) respectively [56, 63], while Huang et al. developed their own survey to quantify self-efficacy [29]. Only Huang et al. and Kiyoshi-Teo et al. showed an increase in self-efficacy following hospital falls education [29, 56].

Cerilo et al. and Kiyoshi-Teo at al. quantified the level of patient engagement in self-management behaviours using the Patient Activation Measure (PAM) instrument [56, 63]. Neither found significant improvements in patient engagement. Hill et al. [55] reported that patients who received videotaped education were able to name more falls strategies than patients who received written education. They also had higher levels of motivation to carry out falls mitigation strategies compared to the group that received only written education [55]. Participants in the study by Dykes et al. gained knowledge of falls prevention strategies [54] while Kiyoshi-Teo et al. did not find changes in falls prevention behaviours on the Modified Falls Behavioural (M-FaB) scale [56]. Kuhlenschmidt et al. assessed participant willingness to ask for assistance as well as their satisfaction with the falls prevention education program [31]. They did not find any change in willingness to ask for help although their satisfaction with the program was high [31].

#### Fall-related outcomes

The fall-related outcomes varied. Most studies analysed falls rates per 1000 patient days in control and experimental groups [30, 32, 38, 52–54, 65, 71, 73, 74, 76]. Others either reported the raw number of falls in intervention and control groups [35, 52, 53, 56, 69–71, 75, 76], falls risk ratio (ratio of cumulative fall incidence in intervention group to control group) [30, 34, 38, 52], falls rate ratio (ratio of fall incidence rate in intervention group to control group) [38, 53, 57], or odds ratio (ratio of the odds of falling in the presence or absence of the intervention) [38, 65]. The injury rates associated with falls were sometimes given [28, 32, 38, 54, 68, 70, 71, 73]. Most of the trials showed an improvement in falls outcomes, with seven reporting otherwise [28, 30, 52, 53, 56, 66, 71]. Other outcomes included the impact of falls prevention interventions on staff knowledge and practice [31, 58, 71], patient adherence to the falls prevention interventions [32, 61, 69], changes in gait and balance [66], and economic outcomes [64, 68, 69]. Overall, the literature showed positive benefits of hospital falls education.

A sub-group analysis of patients with cognitive impairment was performed in two trials [30, 38]. While Haines et al. [30] found similar rates of falls overall between patient education intervention and control groups, cognitively impaired participants had a higher rate of falls with injury compared with the control group. Hill et al. [38] found that patients with cognitive impairment had less reduction in falls compared to those with intact cognition.

### Quality of falls education programs

The studies reviewed scored comparatively low (*n* = 19) or moderately low on quality appraisals (*n* = 12) (Additional file 6). None had a high rating on the patient education quality metric tool. Although most of the education programs had clear aims and purposes, there was seldom recognition of the learner’s prior knowledge or experience. Many did not state if the clinicians or teachers were qualified in teaching or whether they had received training on the falls education program. Several studies included descriptions of the patient learning activities, although only a few assessed the learner’s knowledge or skills post intervention [29, 31, 35, 54–56, 61, 63, 73]. One investigation described evaluating the process of the falls prevention education program by assessing patient satisfaction with the program [31]. Hill et al. [60] published an evaluation of patient awareness, knowledge and confidence to engage in falls prevention strategies after they received education whilst in hospital [38]. None of the remaining trials planned an evaluation of the education program, such as seeking feedback from participants.

###  Systematic reviews

Five systematic reviews examined falls prevention interventions in different hospital settings (Table 1). The settings included acute, sub-acute, rehabilitation, community and residential care facilities. All systematic reviews evaluated multifactorial interventions that incorporated a component of patient education. Two assessed patient education as a single intervention [14, 37]. Each systematic review used a different critical appraisal tool. Three concluded that multifactorial interventions may reduce falls rates [14, 47, 49], whereas one was not able to determine if patient falls education alone was effective [14]. One systematic review of 26 studies that was conducted in 2014 concluded that patient education alone or as a multifactorial intervention reduced falls rates [37].

## Discussion

This scoping review showed patient education to be an important part of falls prevention in hospitals, whether given as a single intervention or delivered within a multifactorial fall mitigation context. Several knowledge gaps were identified. Most notably, many of the identified studies had a minimal focus on educational design and the quality of education. Many were not designed according to evidence-based educational principles or learning theories. Few engaged patients in active learning, which is argued to be associated with gaining a deeper level of understanding and higher engagement [81].

Some links were found between the quality of education programs and a reduction in falls and fall-related injuries. Twenty-eight trials assessed falls-related outcomes and eight of these were RCTs with level II evidence [82], three of which scored moderate for patient education quality [30, 38, 56]. Of these, Hill et al. [38] achieved a significant reduction in falls post-education. However, Haines et al. [30] and Kiyoshi-Teo et al. [56] did not find a difference in falls rates and monthly incidence rates following patient education. Hill et al. [38] reported teacher characteristics and provided age-appropriate learning activities for patients as well as specific falls education content. The trial design used by Hill et al. [38] also appeared to facilitate a growing safety culture in the ward and was developed specifically for hospital inpatients. The content focused on encouraging patients to interact with staff who could have provided reinforcement for the learning that occurred. The use of videotapes delivered on screens and with headphones aimed to assist patients with visual and auditory impairments. This may have increased the uptake of falls prevention strategies as falls rates were reduced across whole units. These units included patients with impaired cognition who did not receive personalised falls education.

The finding that patient education reduces hospital falls was also evident in many of the non-RCT studies [82]. Those which scored high on the quality metric appeared to be more effective in reducing falls-related outcomes, regardless of whether the intervention was single or multifactorial. For example, Martin [61] trialled patient education as a single intervention. They utilised a validated model of the “teach-back” method [83] and found that falls were reduced post-intervention. The trial by Quigley et al. [73] conducted falls education using “teach-back” which was part of a multifactorial intervention and was reported to reduce hospital falls rates.

For the remaining studies, there was a trend towards multifactorial interventions with a component of targeted patient education being most helpful. Five of these were RCTs, and three of them reported a statistically significant reduction in hospital falls rates with multifactorial interventions [32, 34, 57]. This trend was also reflected in non-RCT studies, most of which trialled patient education as part of a multifactorial bundle (Additional file 7). These data need to be interpreted cautiously due to the heterogeneity in education modes, environments and hospital types. It is difficult to ascertain the influence of the patient education component alone within multifactorial interventions.

Few trials applied educational theory, educational principles or a patient engagement framework to inform the design of patient education programs. Incorporating these factors has been shown to optimise health education in other chronic diseases, such as heart failure [84, 85] and cancer [86, 87]. Engaging participants in active learning can also be advantageous [81]. By actively participating in the learning process, patients are more likely to improve their self-efficacy and level of knowledge about falls prevention [88]. Adults are intrinsically motivated to learn, and education can improve their self-perceived falls risk and promote positive changes in health behaviours [89, 90]. Applying health behaviour change models with adequate descriptions is therefore recommended when designing and implementing hospital falls prevention programs [91–93].

The literature that we reviewed suggested that patients can sometimes experience feelings of stress or loss of control during their hospitalisation [94, 95]. This has the potential to affect their ability to process and retain new information [94, 95], such as how to prevent falls in different contexts. When designing new falls prevention programs for hospital patients, it seems important to consider the context, task demands and individual needs.

A recurring theme in the literature that we reviewed was that cognitive impairment can have an adverse effect on the ability of patients to prevent falls [30, 38]. The design and modification of patient education programs for people with cognitive impairment needs careful consideration. A study by Kiegaldie et al. [96] illustrated the challenges associated with delivering education to people with cognitive impairment. They recommended the use of specific techniques such as “chunking”, repetition, simplification, rephrasing, using concrete examples/stories and frequent positive reinforcement when designing education programmes for patients with cognitive impairment [46, 96]. The overall message is that more research is needed on how to modify existing falls education programmes to these patients and how best to measure educational outcomes when cognition is impaired.

There were some limitations of this review, such as not including articles that were published in languages other than English and the exclusion of paediatric and non-hospital populations. Not all trials gave falls rates, and some only conducted pre-post analyses on falls-related outcomes. We found numerous falls prevention strategies for use in hospitals yet many had low levels of supporting evidence [14]. In a recent Cochrane systematic review, Cameron et al. [14] reported the quality of most studies on hospital falls prevention to be low. Although that review concluded that some multifactorial interventions and some single methods may reduce falls rates in hospitals, further high quality controlled clinical trials are needed to verify whether this is always the case. Few investigations explored whether physically restricting mobility could reduce hospital falls, possibly due to ethics concerns pertaining to physical restraints. A strength of this review was the scoping methodology [42–44] which allowed a broad examination of the literature to identify and clarify key concepts in hospital falls prevention education [41, 43].

## Conclusion

Evidence is accumulating that hospital falls prevention interventions that include patient education have the potential to reduce falls. Although no single model of patient education was found to be effective for every person, this scoping review identified key themes. These were: (i) the design and delivery of falls education needs to take into account individual falls risks and environmental context [38]; (ii) a combination of education modes (e.g. face to face discussions, handouts, videotapes) can sometimes be more effective than using a single modality, although this varies according to hospital settings and patient characteristics; (iii) falls education interventions are most helpful when their design incorporates theories of health behaviour change and educational principles; (iv) incorporation of an active learning design can better engage some patients. In addition, reporting of interventions should follow established guidelines to ensure transparency and improve the quality of hospital falls research [8, 97].

## Supplementary information

**Additional file 1.** Search strategy for CINAHL. Example of search strategy used in the literature database of CINAHL.

**Additional file 2.** Modified quality metric of education design. Modified quality metric tool used to assess the quality of education program design.

**Additional file 3.** PRISMA flow diagram of search results. PRISMA flow diagram of search results.

**Additional file 4.** Table of included studies. Characteristics of all included studies in this scoping review.

**Additional file 5.** Table of content, delivery and design of each education program. Descriptive characteristics of content, delivery and design of each education program in included studies.

**Additional file 6.** Quality metric scores of patient education programs. Quality metric scores of each patient education program presented in included studies.

**Additional file 7.** Characteristics of non-RCT studies. Descriptive characteristics of all non-RCT studies including setting, intervention type, patient education content, delivery, design, outcomes and quality.

## Data Availability

The datasets used and/or analysed during the current study are available from the corresponding author on reasonable request.

## Abbreviations

NICE: National Institute for Health and Care Excellence
FRAT: Falls Risk Assessment Tool
STRATIFY: St Thomas’s Risk Assessment Tool
PRISMA-ScR: Preferred Reporting Items for Systematic Reviews and Meta-analysis Extension for Scoping Reviews
CINAHL: Cumulative Index to Nursing and Allied Health Literature
MeSH: Medical subject headings
AMED: Allied and Complementary Medicine Database
CENTRAL: Cochrane Central Register of Controlled Trials
ERIC: Education Resources Information Center
RCT: Randomised Controlled Trial
ProFaNE: Prevention of Falls Network Europe
FRAQ: Falls Risk Awareness Questionnaire
FES: Falls Efficacy Scale
FESI-S: Falls Efficacy Scale International-Short
PAM: Patient Activation Measure
M-FaB: Modified Falls Behavioural scale
