# Supplementary material for: Hospital falls prevention with patient education: a scoping review
Source: BMC Geriatr. 2020 Apr 15;20:140. doi: 10.1186/s12877-020-01515-w (PMC7161005; doi:10.1186/s12877-020-01515-w)
Supplement: Supplementary file 1 — Additional file 1. Search strategy for CINAHL. Example of search strategy used in the literature database of CINAHL. [file 12877_2020_1515_MOESM1_ESM.docx]

Additional file 1: Search strategy for CINAHL

| **#** | **Query** | **Limiters/Expanders** | **Results** |
| --- | --- | --- | --- |
| S11 | S4 AND S9 | Limiters - Published Date: 20080101-20191231; Age Groups: All Adult; Language: English  Search modes - Boolean/Phrase | 4,421 |
| S10 | S4 AND S9 | Search modes - Boolean/Phrase | 16,642 |
| S9 | S5 OR S6 OR S7 OR S8 | Search modes - Boolean/Phrase | 96,075 |
| S8 | patient education OR patient education handout OR Carers education OR Carer education OR Caregiver education OR Care Givers education OR Spouse Caregivers education OR Spouse Caregiver education OR Family Caregivers education OR Family Caregiver education OR Hospital falls OR patient falls OR Reducing fall OR reduce fall OR reduce falls OR reducing falls OR falls reduction OR fall reduction OR reduced falls OR reduced fall OR Fallers OR Fall prevention OR Falls prevention OR Preventing falls OR Preventing fall OR prevent falls OR fall rates OR recurrent fall OR falls intervention OR falls prevention intervention OR inpatient fall* | Search modes - Boolean/Phrase | 96,075 |
| S7 | (MH "Accidental Falls/PC") | Search modes - Boolean/Phrase | 8,832 |
| S6 | (MH "Caregivers/ED") | Search modes - Boolean/Phrase | 2,172 |
| S5 | (MH "Patient Education") | Search modes - Boolean/Phrase | 59,809 |
| S4 | S1 OR S2 OR S3 | Search modes - Boolean/Phrase | 484,915 |
| S3 | Inpatient* OR Hospitals OR private hospital* OR geriatric hospital* OR public hospital* OR teaching hospital* OR general hospital* OR aged hospital* OR community hospital* OR university hospital* OR acute hospital* OR subacute hospital* OR sub-acute hospital* OR rehab* hospital* OR community hospital* OR rural hospital* OR urban hospital* | Search modes - Boolean/Phrase | 484,915 |
| S2 | (MH "Hospitals") | Search modes - Boolean/Phrase | 53,115 |
| S1 | (MH "Inpatients") | Search modes - Boolean/Phrase | 76,177 |
