# Supplementary material for: Hospital falls prevention with patient education: a scoping review
Source: BMC Geriatr. 2020 Apr 15;20:140. doi: 10.1186/s12877-020-01515-w (PMC7161005; doi:10.1186/s12877-020-01515-w)
Supplement: Supplementary file 2 — Additional file 2. Modified quality metric of education design. Modified quality metric tool used to assess the quality of education program design. [file 12877_2020_1515_MOESM2_ESM.docx]

Additional file 2: Modified quality metric of education design

| **Item** | **Key questions** |
| --- | --- |
| **Purpose / Aim** | Is the purpose and rationale of the education program stated?  Is there a clear direction to the program?  Is there a satisfactory description of the significance of the program?  Is the education conducted in a suitable setting |
| **Learner/Co-Learner characteristics** | Is the program pitched towards an appropriate audience?  Is there recognition of learner’s/co-learner’s prior knowledge/experience? |
| **Teacher characteristics** | Is there a description of who is teaching the program?  Are the teachers qualified and/or experienced on the topic?  Are the teachers qualified and/or experienced in teaching?  Is training on the program offered? |
| **Learning activities** | Is there description of the learning activities?  Are the learning activities suitable for supporting learners/co-learners to meet the learning objectives?  Is there an assessment of learner’s/co-learner’s achievement of learning objectives (knowledge, skills, attitudes) |
| **Education evaluation** | Has an evaluation been planned?  Is the evaluation method appropriate?  Has an evaluation been conducted?  Are the education outcomes reported for process (learner’s/ co-learner’s views on the teaching) |
