# Supplementary material for: Hospital falls prevention with patient education: a scoping review
Source: BMC Geriatr. 2020 Apr 15;20:140. doi: 10.1186/s12877-020-01515-w (PMC7161005; doi:10.1186/s12877-020-01515-w)
Supplement: Supplementary file 3 — Additional file 3. PRISMA flow diagram of search results. PRISMA flow diagram of search results. [file 12877_2020_1515_MOESM3_ESM.docx]

Additional File 3: PRISMA flow diagram of search results

Records identified through searching multiple databases (n= 9340)

Additional records identified through other sources (n= 41)

Records after duplicates removed (n= 8061)

Records screened
(n= 8061)

Records excluded (n= 7950)

Full-text articles to be assessed for eligibility
(n = 111)

Full-text articles were excluded
(n = 73)

Unable to retrieve full text (n = 8)
Protocol paper (n = 9)
Wrong population (n = 2)
No patient education (n = 47)
Non-empirical study (n = 7)

Studies included in synthesis (n = 43)

Records included from hand searching identified systematic and non-systematic reviews (n = 5)
