# Supplementary material for: Hospital falls prevention with patient education: a scoping review
Source: BMC Geriatr. 2020 Apr 15;20:140. doi: 10.1186/s12877-020-01515-w (PMC7161005; doi:10.1186/s12877-020-01515-w)
Supplement: Supplementary file 4 — Additional file 4. Table of included studies. Characteristics of all included studies in this scoping review. [file 12877_2020_1515_MOESM4_ESM.docx]

Additional file 4: Included studies

| Lead author | Year | Article type | Study design | Falls prevention intervention |
| --- | --- | --- | --- | --- |
| Aizen | 2015 | Quantitative study | RCT | Multifactorial* v Usual Care |
| Ang | 2011 | Quantitative study | RCT | Multifactorial* v Usual Care |
| Avanecean | 2017 | Review | Systematic Review | N/A |
| Beasley | 2009 | Quantitative study | Quasi-experimental | Multifactorial* |
| Cameron | 2018 | Review | Systematic Review | N/A |
| Cangany | 2015 | Quantitative study | Quasi-experimental | Multifactorial* |
| Cerilo | 2016 | Thesis | Quasi-experimental | Education of patient |
| Clarke | 2011 | Quantitative study | Quasi-experimental | Education of patient preadmission v Usual care |
| Cumming | 2008 | Quantitative study | RCT | Multifactorial* v Usual Care |
| Dacenko-Grawe | 2008 | Quantitative study | Quasi-experimental | Multifactorial* |
| Dykes | 2017 | Quantitative study | Quasi-experimental | Multifactorial v Usual Care |
| Dykes | 2010 | Quantitative study | RCT | Multifactorial* v Usual Care |
| Forrest | 2012 | Quantitative study | Quasi-experimental | Multifactorial* |
| Haines | 2011 | Quantitative study | RCT | Education of patient v Usual Care |
| Haines | 2013 | Quantitative study | Economic evaluation | N/A |
| Hempel | 2013 | Review | Systematic Review | N/A |
| Hill | 2015 | Quantitative study | RCT | Education of patient v Usual Care |
| Hill | 2016 | Qualitative study | Qualitative study | N/A |
| Hill | 2015 | Qualitative study | Qualitative study | N/A |
| Hill | 2016 | Qualitative study | Qualitative study | N/A |
| Hill | 2009 | Quantitative study | RCT | Education of patient delivered by video v Education of patient delivered by handout |
| Huang | 2015 | Quantitative study | Quasi-experimental | Education of patient v Usual Care |
| Khalifa | 2019 | Review | Targeted literature review | N/A |
| Kiyoshi-Teo | 2019 | Quantitative study | RCT | Education of patient v Usual Care |
| Kobayashi | 2017 | Quantitative study | Quasi-experimental | Multifactorial* |
| Kolin | 2010 | Quantitative study | Quasi-experimental | Multifactorial* |
| Krauss | 2008 | Quantitative study | Quasi-experimental | Multifactorial* |
| Kuhlenschmidt | 2016 | Quantitative study | RCT | Education of patient v Usual Care |
| Lee | 2014 | Review | Systematic Review | N/A |
| LeLaurin | 2019 | Review | Targeted literature review | N/A |
| Martin | 2017 | Thesis | Quasi-experimental | Education of patient |
| Miake-Lye | 2013 | Review | Systematic Review | N/A |
| Miller | 2008 | Quantitative study | Quasi-experimental | Multifactorial* |
| Oliver | 2010 | Review | Targeted literature review | N/A |
| Quigley | 2009 | Quantitative study | Quasi-experimental | Multifactorial* |
| Shuey | 2014 | Quantitative study | Quasi-experimental | Multifactorial* |
| Sitzer | 2014 | Thesis | Quasi-experimental | Education of patient |
| Stoeckle | 2019 | Quantitative study | Quasi-experimental | Multifactorial* |
| Trombetti | 2013 | Quantitative study | Quasi-experimental | Multifactorial* |
| van Gaal | 2010 | Quantitative study | RCT | Multifactorial* v Usual Care |
| Vieira | 2013 | Quantitative study | Quasi-experimental | Multifactorial* v Usual Care |
| Wayland | 2010 | Quantitative study | Quasi-experimental | Multifactorial* |
| Zavotsky | 2014 | Quantitative study | Quasi-experimental | Education of patient |

RCT = Randomised controlled trial; N/A = not applicable* refers to one or more of the following: falls risk assessments, environmental modifications, personal supervision, multidisciplinary reviews, medication reviews, falls risk communication aids, allied health input, rounding, staff training

Footnote: Qualitative studies were not included in the table due to differing outcomes.
