# Supplementary material for: Hospital falls prevention with patient education: a scoping review
Source: BMC Geriatr. 2020 Apr 15;20:140. doi: 10.1186/s12877-020-01515-w (PMC7161005; doi:10.1186/s12877-020-01515-w)
Supplement: Supplementary file 5 — Additional file 5. Table of content, delivery and design of each education program. Descriptive characteristics of content, delivery and design of each education program in included studies. [file 12877_2020_1515_MOESM5_ESM.docx]

Additional file 5: Table of content, delivery and design of each education program

| First author (year) | Content | | Delivery | | | | Design |
| --- | --- | --- | --- | --- | --- | --- | --- |
|  | **Falls risk** | **Falls prevention strategies** | **Face to face** | **Videotaped** | **Posters** | **Handouts** | **Guiding principles?** |
| Aizen (2015) | NS | NS |  |  |  |  | NS |
| Ang (2011) | Y | Y | ✓ |  |  |  | NS |
| Beasley (2009) | Y | Y | ✓ |  |  | ✓ | NS |
| Cangany (2015) | NS | NS | ✓ |  | ✓ |  | NS |
| Cerilo (2016) | Y | Y | ✓ | ✓ |  |  | NS |
| Clarke (2011) | Y | Y | ✓ |  |  |  | Y  Teach-back method |
| Cumming (2008) | Y | Y | ✓ |  |  |  | NS |
| Dacenko-Grawe (2008) | NS | Y |  |  | ✓ | ✓ | NS |
| Dykes (2010) | Y | Y |  |  |  | ✓ | Y  Consumer literacy |
| Dykes (2017) | Y | Y |  |  |  | ✓ | Y  Consumer literacy |
| Forrest (2012) | NS | Y | ✓ |  |  |  | NS |
| Haines (2011) | NS | NS | ✓ | ✓ |  | ✓ | Y  HBM |
| Hill (2009) | Y | Y |  | ✓ |  | ✓ | Y  HBM, design and communication principles |
| Hill (2015) | Y | Y | ✓ | ✓ |  | ✓ | Y  HBM, ALP |
| Huang (2015) | Y | NS | ✓ |  |  | ✓ | Y  ALP |
| Kiyoshi-Teo (2019) | Y | Y | ✓ |  |  | ✓ | Y  Motivational interviewing |
| Kobayashi (2017) | Y | NS |  | ✓ | ✓ | ✓ | Y  ALP |
| Kolin (2010) | NS | NS |  | ✓ |  | ✓ | NS |
| Krauss (2008) | Y | Y | ✓ |  |  | ✓ | NS |
| Kuhlenschmidt (2016) | Y | Y | ✓ | ✓ |  | ✓ | NS |
| Martin (2017) | Y | Y | ✓ | ✓ |  |  | Y  Teach-back method |
| Miller (2008) | NS | Y | ✓ |  | ✓ |  | NS |
| Quigley (2009) | Y | Y | ✓ |  |  |  | Y  Teach-back method |
| Shuey (2014) | Y | Y | ✓ |  |  | ✓ | NS |
| Sitzer (2014) | Y | Y |  | ✓ |  |  | Y  Patient engagement framework |
| Stoeckle (2019) | NS | NS | ✓ |  |  | ✓ | NS |
| Trombetti (2013) | Y | Y | ✓ |  |  |  | NS |
| van Gaal (2010) | NS | NS |  |  |  | ✓ | NS |
| Vieira (2012) | Y | Y |  |  |  | ✓ | NS |
| Wayland (2010) | NS | Y |  |  | ✓ |  | NS |
| Zavotsky (2014) | Y | Y | ✓ |  |  | ✓ | NS |

Y: Yes; NS: Not stated; HBM: Health Belief Model; ALP: Adult Learning Principles
