# Supplementary material for: Hospital falls prevention with patient education: a scoping review
Source: BMC Geriatr. 2020 Apr 15;20:140. doi: 10.1186/s12877-020-01515-w (PMC7161005; doi:10.1186/s12877-020-01515-w)
Supplement: Supplementary file 6 — Additional file 6. Quality metric scores of patient education programs. Quality metric scores of each patient education program presented in included studies. [file 12877_2020_1515_MOESM6_ESM.docx]

Additional file 6: Quality metric scores of patient education programs

| Lead Author (Year) | Purpose/Aim (/4) | Learner Characteristics (/2) | Teacher Characteristics (/4) | Learning activities (/3) | Evaluation (/4) | Total (/17) |
| --- | --- | --- | --- | --- | --- | --- |
| Aizen (2015) | 1 | 1 | 0 | 0 | 0 | 2 Low |
| Ang (2011) | 3 | 1 | 2 | 0 | 0 | 6 Low |
| Beasley (2009) | 4 | 1 | 3 | 0 | 0 | 8 Mod |
| Cangany (2015) | 1 | 1 | 1 | 2 | 0 | 5 Low |
| Cerilo (2016) | 4 | 1 | 1 | 2 | 0 | 8 Mod |
| Clarke (2011) | 4 | 1 | 2 | 1 | 0 | 8 Mod |
| Cumming (2008) | 2 | 1 | 2 | 0 | 0 | 5 Low |
| Dacenko-Grawe (2008) | 2 | 1 | 2 | 0 | 0 | 5 Low |
| Dykes (2010) | 3 | 1 | 0 | 0 | 0 | 4 Low |
| Dykes (2017) | 3 | 1 | 0 | 1 | 0 | 5 Low |
| Forrest (2012) | 2 | 1 | 2 | 0 | 0 | 5 Low |
| Haines (2011) | 4 | 1 | 2 | 0 | 0 | 7 Mod |
| Hill (2009) | 4 | 1 | 0 | 3 | 0 | 8 Mod |
| Hill (2015) | 4 | 1 | 4 | 2 | 0 | 11 Mod |
| Huang (2015) | 2 | 1 | 3 | 3 | 0 | 9 Mod |
| Kiyoshi-Teo (2019) | 2 | 2 | 3 | 3 | 0 | 10 Mod |
| Kobayashi (2017) | 1 | 0 | 0 | 1 | 0 | 2 Low |
| Kolin (2010) | 2 | 1 | 0 | 0 | 0 | 3 Low |
| Krauss (2008) | 1 | 1 | 3 | 0 | 0 | 5 Low |
| Kuhlenschmidt (2016) | 3 | 2 | 3 | 1 | 2 | 11 Mod |
| Martin (2017) | 4 | 1 | 3 | 3 | 0 | 11 Mod |
| Miller (2008) | 1 | 1 | 0 | 0 | 0 | 2 Low |
| Quigley (2009) | 2 | 1 | 2 | 3 | 0 | 8 Mod |
| Shuey (2014) | 3 | 0 | 0 | 0 | 0 | 3 Low |
| Sitzer (2014) | 3 | 1 | 0 | 0 | 0 | 4 Low |
| Stoeckle (2019) | 2 | 1 | 2 | 0 | 0 | 5 Low |
| Trombetti (2013) | 3 | 1 | 2 | 0 | 0 | 6 Low |
| van Gaal (2010) | 1 | 1 | 0 | 0 | 0 | 2 Low |
| Vieira (2012) | 1 | 1 | 0 | 0 | 0 | 2 Low |
| Wayland (2010) | 2 | 1 | 0 | 0 | 0 | 3 Low |
| Zavotsky (2014) | 4 | 1 | 2 | 2 | 0 | 9 Mod |
