# Supplementary material for: Hospital falls prevention with patient education: a scoping review
Source: BMC Geriatr. 2020 Apr 15;20:140. doi: 10.1186/s12877-020-01515-w (PMC7161005; doi:10.1186/s12877-020-01515-w)
Supplement: Supplementary file 7 — Additional file 7. Characteristics of non-RCT studies. Descriptive characteristics of all non-RCT studies including setting, intervention type, patient education content, delivery, design, outcomes and quality. [file 12877_2020_1515_MOESM7_ESM.docx]

Additional file 7: Characteristics of non-RCT studies

| Lead author (Year) | Setting | Interventions | Patient education content | Education delivery modes | Education design guiding principles? | Education outcomes | Education quality | Fall outcomes |
| --- | --- | --- | --- | --- | --- | --- | --- | --- |
| Beasley (2009) | Acute | Multifactorial* | Education on medication related falls risk and reinforcement of nursing care plan | Face to face and handouts by pharmacists | Not stated | Not reported | 8/17 Moderate | Reduced falls rate (from 5 falls per 1000 patient days to 4 falls per 1000 patient days) |
| Cangany (2015) | Acute | Multifactorial* | Not reported | Face to face by nurses | Not stated | Not reported | 5/17 Low | Reduced falls (from 37 falls in 2011 to 11 falls in 2013) |
| Cerilo (2016) | Acute | Education of patient | Falls education based on individual falls risk | Face to face and video by research nurses | Not stated | Yes. Increased fall risk awareness (19.02 on FRAQ baseline to 21.71 post intervention). No difference in falls self-efficacy and patient engagement. | 5/17 Low | Not reported |
| Clarke (2011) | Acute | Education of patient preadmission v Usual care | Falls education based on falls risks specific to post-op TKA. Patient required to recount falls prevention strategies | Face to face and handout by nurses | Not stated | Not reported | 8/17 Moderate | Less falls in intervention than control group (0 falls intervention; 7 falls control) (p=0.03) |
| Dacenko-Grawe (2008) | Acute | Multifactorial* | Education on falls preventions strategies | Handout by nurses and posters | Not stated | Not reported | 5/17 Low | Reduced falls rate (from 4 falls per 1000 patient days to 2 falls per 1000 patient days) |
| Dykes (2017) | Acute | Multifactorial v Usual Care | Falls education based on individual falls risks | Handout | Yes. Handout designed to match consumer literacy | Yes. Increased awareness of falls risk (4.0 on Likert scale baseline to 4.6 post intervention) and falls prevention strategies (3.6 on Likert scale baseline to 4.7 post intervention). | 5/17 Low | Reduced falls rate (from 3.28 per 1000 patient days to 2.80 per 1000 patient days) |
| Forrest (2012) | Sub-acute | Multifactorial* | Education on risk of falls and falls prevention strategies | Face to face by nurses | Not stated | Not reported | 5/17 Low | Reduced number of fallers (from 12.5% of patients falling in 2006 to 7.3% in 2009) |
| Huang (2015) | Acute | Education of patient v Usual Care | Education on fall risks, fall strategies and fear of falling, tailored to different risk categories | Face to face by researcher and health educator | Yes. Content based on adult learning theory. | Yes. Increased falls and falls prevention knowledge (p<0.001) and self-efficacy (p<0.01) post intervention. | 8/17 Moderate | Reduced falls rate (0% per 100 patient days intervention; 19.3% per 100 patient days control). |
| Kobayashi (2017) | Acute | Multifactorial* | Education on falls and risk of falls | Face to face plus handout given by nurses | Not stated | Not reported | 2/17 Low | Reduced falls (incidence rate from 2.1% in 2012 to 1.3% in 2016 p<0.01) |
| Kolin (2010) | Acute | Multifactorial* | Education on falls | Handout and video given by staff | Not stated | Not reported | 3/17 Low | Reduced falls post intervention |
| Krauss (2008) | Acute | Multifactorial* | Education on falls risk and falls prevention strategies | Face to face and handout by staff | Not stated | Not reported | 5/17 Low | No difference in falls rate (5 falls per 1000 patient days post intervention; 7 falls per 1000 patient days pre intervention) (p=0.182) |
| Martin (2017) | Acute | Education of patient | Education on falls prevention strategies | Face to face and video by nurses | Not stated | Not reported | 11/17 Moderate | Reduced falls post intervention |
| Miller (2008) | Acute | Multifactorial* | No description | Handout by nurses | Not stated | Not reported | 2/17 Low | Reduced fall index 6 months post intervention |
| Quigley (2009) | Acute | Multifactorial* | Falls education based on individualised falls risk | Face to face by nurses | Not stated | Not reported | 8/17 Moderate | Reduced falls rate post intervention (from 4 falls per 1000 patient days to 3 falls per 1000 patient days) |
| Shuey (2014) | Acute | Multifactorial* | Education on falls risk and falls prevention strategies | Handout by nurses | Not stated | Not reported | 3/17 Low | Reduced falls post intervention |
| Sitzer (2014) | Acute | Education of patient | Education on falls risk and falls prevention strategies | Interactive video | Yes. Content based on patient engagement framework | Not reported | 4/17 Low | Each additional prompt to watch the video was associated with an increase in the odds of a fall by 1.5 |
| Stoeckle (2019) | Acute | Multifactorial* | Not reported | Face to face and handout by nurses | Not stated | Not reported | 5/17 Low | No difference in falls |
| Trombetti (2013) | Acute, sub-acute | Multifactorial* | Education on falls risk and prevention strategies | Face to face by occupational therapist in groups | Not stated | Not reported | 6/17 Low | No difference in falls |
| Vieira (2013) | Sub-acute | Multifactorial* v Usual Care | Education on falls risk and prevention strategies | Handout | Not stated | Not reported | 2/17 Low | Reduced falls rate post intervention (from 7 falls per 1000 patient days to 4 falls per 1000 patient days) |
| Wayland (2010) | Acute, sub-acute | Multifactorial* | No description | Face to face and posters | Not stated | Not reported | 3/17 Low | Reduced falls post intervention (from 4 falls per 1000 patient days to 1 fall per 1000 patient days) |
| Zavotsky (2014) | Acute | Education of patient | Education on falls risk and prevention strategies | Face to face and handout by nurses | Not stated | Not reported | 9/17 Moderate | No difference in falls |

* refers to one or more of the following: falls risk assessments, environmental modifications, personal supervision, multidisciplinary reviews, medication reviews, falls risk communication aids, allied health input, rounding, staff training

Footnote: Qualitative studies were not included in the table due to differing outcomes.
